# ASAS-NANP symposium: mathematical modeling in animal nutrition: application of modeling innovations to support satellite remote sensing for sustainable grazing cattle management

**DOI:** 10.1093/jas/skaf137

**Published:** 2025-05-04

**Authors:** Marcia Helena Machado da Rocha Fernandes, Luis Orlindo Tedeschi

**Affiliations:** Department of Animal Science, Sao Paulo State University, Jaboticabal, SP 14884-900, Brazil; Department of Animal Science, Texas A&M University, College Station, TX 77845, USA; Department of Animal Science, Texas A&M University, College Station, TX 77845, USA

**Keywords:** cattle, grazing, modelling, nutrition systems, satellite

## Abstract

Integrating modeling innovations and satellite remote sensing technology offers a transformative approach to sustainable grazing cattle management. Mathematical models, which translate real-life situations into mathematical formulations, are becoming critical components in livestock production, especially for describing patterns and predicting behaviors. Mathematical models are categorized by their purpose and methodology and include descriptive, prescriptive, static, dynamic, deterministic, and stochastic types. Grazing lands, covering 24.6% of the world’s land area, provide essential ecosystem services such as soil stability, nutrient cycling, and climate regulation. Sustainable management of these lands is necessary to optimize grazing performance and prevent degradation. Given its ability to rapidly scan vast expanses, satellite remote sensing has become indispensable for monitoring grassland conditions over large areas, surpassing traditional field methods in coverage and efficiency. Modeling approaches using satellite imagery include parametric and nonparametric artificial intelligence-based regression and physically based models. Parametric models, such as those based on vegetation indices, offer simplicity but may struggle with high vegetation cover and soil background interference. Nonparametric models, including machine learning algorithms like random forest and support vector regression, provide flexibility and improved accuracy in estimating forage mass and nutritional attributes. Physically based models, like canopy radiation transfer models, integrate satellite data to simulate vegetation dynamics. Practical applications of satellite-based vegetation data support real-time, continuous grazing management by adjusting stocking rates and predicting average daily gain. Studies demonstrate that integrating satellite data with field observations and mechanistic models can optimize forage use, improve livestock productivity, and enhance the sustainability of grazing systems. This comprehensive review highlights the pivotal role of satellite remote sensing in revolutionizing grazing cattle management, providing a detailed exploration of the technologies and models that drive sustainable practices in this field. Through continuous advancements, satellite-based approaches promise to enhance precision livestock farming further, contributing to ecological and economic sustainability.

## Teaser

Integrating modeling innovations and satellite remote sensing technology offers a transformative approach to sustainable grazing cattle management. This comprehensive review highlights the pivotal role of satellite remote sensing in revolutionizing grazing cattle management, providing a detailed exploration of the technologies and models that drive sustainable practices in this field.

## Introduction

In animal science, modeling has emerged as a pivotal tool for advancing our understanding of managing complex biological systems and predicting outcomes (Jacobs et al., 2022). Integrating mathematical and computational models in animal science research offers a potent approach to dissecting intricate interactions within and between animal populations, environments, and management practices (Tedeschi, 2019). Conceptually, mathematical models (**MM**) are defined as “*mental conceptualizations, enclosed in a virtual domain, whose purpose is to translate real-life situations into mathematical formulations (symbolically or numerically) to describe existing patterns or forecast future behaviors in the real-life situations*” (Tedeschi, 2019).

Nonetheless, Jacobs et al. (2022) pointed out that the perception of a model representation depends on the individual scientific point of view. For instance, a model for a mathematician is made up of formulas, while for a social scientist, a model can be a textual mental conceptualization (Jacobs et al., 2022). Also, the categorization of models has broad ways, depending on scope and purpose, such as descriptive (i.e., elucidative) or prescriptive (i.e., predictive), static (i.e., steady state, prediction of a single time point) or dynamic (i.e., considering changes over time), deterministic (considering the “average” animal) or stochastic (i. e., including probabilistic variation), empirical (describe a simple correlation in the data) or mechanistic (i. e., underlying casual pathways) (Tedeschi, 2019; Jacobs et al., 2022). It is noteworthy that hybrid MM exists as a combination of different categories.

Therefore, discussing model innovations in animal science depends on the field or area of animal agriculture, offering substantial benefits across various domains, for instance, in the sustainable management of grazing cattle through satellite remote sensing. Modeling innovations in animal science play a critical role in enhancing productivity, improving nutrition, and promoting sustainable practices (Tedeschi, 2022). Traditional modeling techniques may stagnate if outdated technologies and similar models are used (Tedeschi, 2019; Tedeschi et al., 2021). In the new era of precision livestock farming (**PLF**), MM are an essential component in supporting technologies to enhance animal production (Tedeschi et al., 2021).

### The grazing cattle

Grazing lands comprise 24.6% of land area and 67% of agricultural area worldwide (FAO, 2023). They contribute to maintaining soil stability, nutrient cycling, and natural habitat; regulate the climate, pollinate and purify water; offer cultural and recreational opportunities, ecotourism, and aesthetically pleasant landscapes, among other crucial ecosystem services (Sanderson and Liebig, 2020). In addition, grazing lands play a significant role in regulating global carbon (Silva et al., 2016; Stanley et al., 2018) by being the second primary source (about 30%) of carbon sink after forests (Ali et al., 2014) or by contributing to direct and indirect emissions of greenhouse gases. For instance, grazing land degradation has serious consequences, such as increased carbon emissions (Herrero et al., 2016; Padarian et al., 2022). Furthermore, grazing lands are the livestock industry’s major and cheapest feed source.

The term “grassland” in the context of this review refers to the description of Allen et al. (2011), which implies a broad interpretation of lands committed to being grazed by animals, including a broad type of vegetation (e.g., grasses, legumes and other forbs, and shrubs) that may be a natural or an imposed ecosystem. Furthermore, grasslands are classified as pasturelands when referring to an imposed grazing-land ecosystem or rangelands when referring to lands whose indigenous vegetation is predominantly native herbaceous and shrubby vegetation that supports feed for grazing livestock and wildlife (Allen et al., 2011). The term “grazing management” in this review refers to a set of human activities to manipulate grazing in pursuit of an objective or a set of them (Allen et al., 2011).

Grazing management (as well as soil and climate conditions) determines the quantity and quality of forage, affecting the productivity and the sustainability of grazing ecosystems. Hence, the sustainable management of grassland ecosystems is necessary to optimize grazing performance and avoid land degradation but requires frequent and spatially comprehensive monitoring of grassland conditions, such as vegetation cover, above-ground forage mass, nutrient concentrations (e.g., fiber, nitrogen, and minerals), weedy infestation, among others (Dusseux et al., 2022).

It has been observed throughout the last 40 yr that remote sensing techniques are a feasible substitute for traditional field and laboratory analyses regarding grazing land monitoring (Wachendorf et al., 2017). Remote sensing is a vast field and has been broadly defined by Horning (2019) as “*the science and practice of acquiring information about an object without coming into contact with it*.” The sensors, mostly optical sensors, are available on handheld instruments or mounted in ground vehicles, eddy covariance towers, unperson aerial vehicles **(UAV)**, aircraft, and satellite platforms (Ali et al., 2016).

The application of satellite remote sensing for monitoring grasslands has been used for various purposes over the years, and different equations and models have been developed depending on the purpose and technology available (Tedeschi et al., 2019, 2023; Reinermann et al., 2020). It is important to emphasize that within the context of satellite remote sensing for monitoring grasslands, evolution has occurred by improving the data captured by sensors and refining the models used to interpret this data, ranging from simple linear equations to complex machine learning algorithms.

Thus, this review focused on satellite remote sensing, also known as orbital remote sensing or spaceborne remote sensing, which are platforms used to hold remote-sensing instruments, such as spectral cameras. We presented an overview of the critical concepts and evaluations of existing models used to derive grassland parameters from satellite data, aiming at their application in models for monitoring and managing rangelands and pastures at the paddock level. Specifically, the review highlights the retrieval of above-ground forage mass and chemical composition as critical parameters. In the first part, we briefly described the fundamental principles of satellite remote sensing, showing its capability to capture and analyze data across vast expanses of land. In the second part, we discussed the various equations and mathematical models used to retrieve different grassland parameters (such as above-ground forage mass and chemical composition), addressing some advantages and limitations. In the third and final part, we present an overview of how the grassland information retrieved from satellite remote sensing has been used in more complex decision-making models to assess and assist ranchers in sustainably managing their pastures and rangelands.

## Recent Advances in Satellite Technology

In 1972, the National Aeronautics and Space Administration (NASA) launched its first satellite (Earth Resources Technology Satellite 1, later renamed Landsat 1) and started the Landsat program, which is the longest-running program of satellite remote sensing focused on Earth observation (Horning, 2008). Since the 1970s, satellites used for Earth observation have evolved their sensors and instrumentation package along the exponential technological development, providing more precise and accurate data for use in agricultural, ecological, meteorological, and environmental modeling. Satellites differ in the type of sensor and instrumentation features, such as spectral, radiometric, spatial, and temporal resolutions, which affect the level of detail that can be acquired by a satellite image (Horning, 2019) and, consequently, the ability of satellite-based data to map and monitor biomass and nutritional attributes of grazing lands. For instance, a passive sensor uses solar energy and measures the radiance (the reflected electromagnetic radiation) from features on the Earth’s surface, while an active sensor uses its energy source and measures the strength of the return signal and the time delay between the emitted signal and the returned pulse (Horning, 2019). In this review, we focus on passive or optical sensors.

An image is the most recognizable type of remotely sensed data, which is made up of individual elements arranged in a grid of rows and columns called pixels (Horning, 2019). Thus, spatial resolution refers to the smallest surface area captured by the satellite or the size of the pixel (smallest discrete scene element and image display unit) in ground dimensions and determines the level of detail that can be extracted concerning objects in each scene (Horning, 2019). Temporal resolution refers to the frequency of the data acquisition or how frequently the satellite can capture the same area again, while radiometric resolution refers to the range of digital numbers representing the pixel values (Horning, 2019). Spectral resolution refers to the range of wavelengths detected in a particular image band and the number of bands the sensor can capture. Band placement defines the wavelength range of the electromagnetic spectrum that one image band detects. Multispectral optical sensors measure the reflectance in 3 to 12 wide spectral bands, while hyperspectral sensors acquire data in several hundred very narrow, contiguous spectral bands throughout the spectrum’s visible and near-infrared (**NIR**) portions (Horning, 2019).

Table 1 exemplifies the features of some used optical-sensor satellites in grassland monitoring studies (Reinermann et al., 2020; Ogungbuyi et al., 2023). In general, the Moderate Resolution Imaging Spectroradiometer (**MODIS**; Terra and Aqua) and Landsat satellites were mainly applied in grassland studies (Reinermann et al., 2020; Ogungbuyi et al., 2023), both open source data. MODIS has a high temporal (daily revisit) but low spatial resolution (250 m, 500 m, and 1 km), being primarily used for global, regional, or sub-regional scales (Ogungbuyi et al., 2023). The extensive use of Landsat sensors in grassland studies (Supplementary material) in diverse spatial scales is probably due to its long-term data continuity, moderate-resolution imaging, multispectral capabilities, and open-source data (Ogungbuyi et al., 2023). Nonetheless, the launch of satellite constellations (such as Sentinel-2 and PlanetScope), with finer spatial and temporal resolutions (Table 1) has promoted innovations in pasture management at the paddock level and more frequent intervals (Ogungbuyi et al., 2023).

**Table 1.** Characteristics of satellites with spectral sensor systems

Satellite	Spatial resolution (m)	Temporal resolution (d)	Radiometric resolution (bits)	Spectral resolution (*n* of wavebands)
MODIS	250, 500, 1000	1	12	2, 5, 29
Landsat	15, 30, 100	16	12 to 14	11
Sentinel-2	10, 20, 60	5, 10	12	22
SPOT	2.2, 8.8	1 to 3	8 to 12	5
RapidEye	6.5	1	16	5
PlanetScope	3	1	16	5

Adapted from Wachendorf et al. (2017) and Ogungbuyi et al. (2023).

Abbreviations: MODIS, Moderate Resolution Imaging Spectroradiometer; SPOT, Satellite Pour l’Observation de la Terre.

The Sentinel-2 satellite constellation was launched in 2015 by the European Space Agency, and its data is available as open source. Compared to other open sources of multispectral satellites, such as Landsat and MODIS, Sentinel-2 outperforms in its spectral resolution because of the presence of red-edge bands, which were only previously available in commercial satellites such as WorldView-2 and RapidEye (Ramoelo et al., 2015). The presence of red-edge and short infrared bands in Sentinel-2 has provided an opportunity for assessing not only the above-ground biomass (**AGB**) but also the chemical composition of pastures on a large scale (Mashiane et al., 2023; Zhang et al., 2023; Fernandes et al., 2024).

## Modeling Approaches and Innovations

### Satellite imagery for grazing cattle

Since the beginning of the Landsat project in the 1970s, satellite imagery has been extensively studied and applied in agriculture and ecological monitoring, including diverse applications. Developments in satellite technology, such as improved sensor capabilities and advanced image processing algorithms, have significantly increased the resolution and accuracy of satellite imagery. These improvements enable more detailed monitoring of vegetation health and land use changes over time and harness satellite data as a resourceful tool in PLF (Chizzotti et al., 2022; Heins et al., 2023).

Traditional grazing management often struggles with inefficiencies due to a lack of precise real-time data and predictive capabilities regarding the pasture forage mass and its nutritive value (Tedeschi et al., 2019), whereas “sustainable” grazing practices might be essential for maintaining the ecological balance and promoting the economic viability of livestock farming while improving its ecosystem services (Tedeschi et al., 2024). In this sense, satellite remote sensing has been a cornerstone of PLF to transform traditional grazing system management practices (Chizzotti et al., 2022; Heins et al., 2023). Satellite imagery can provide comprehensive data on pasture forage mass and chemical composition, water availability, and animal movement patterns over extensive areas (Chizzotti et al., 2022; Menendez et al., 2022). Integrating satellite imagery with GIS can provide a detailed analysis of pasture vegetation, directly correlating with the nutritional value of grazing cattle. This information allows for strategic grazing management and supplementation, where cattle are moved to areas with optimal forage quality, thus maximizing the efficiency of pasture usage.

**Figure 1.**
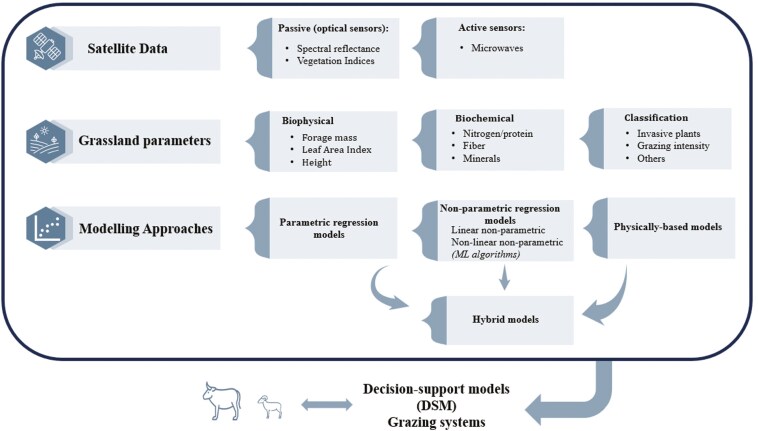
Modelling approaches used to describe the relationship between satellite imagery data and forage parameters.

The first studies relating spaceborne (satellite) spectral indices and above-ground forage mass of grazing lands began in the late 1990s and have shown exponential growth since then (Reinermann et al., 2020). In practical conditions, the great challenge has been to build an MM that precisely and accurately describes and predicts biophysical and biochemical parameters of forages based on physical parameters from satellite data. Fig. 1 depicts the modeling approaches that describe the relationship between satellite imagery data and forage parameters.

We adopted the categorization of models Verrelst et al. (2015) described as parametric versus nonparametricVerrelst et al. (2015) models. In a parametric model, the number of parameters is fixed with respect to the sample size. It is assumed an explicit function that describes the relationship between the response and explanatory variables. Typically, those models rely on statistical and physical knowledge of the variable and the spectral response, being essentially empirical models. Linear, generalized, and nonlinear models are examples of parametric regression models. Unfortunately, the effective number of parameters can grow with the sample size in a nonparametric model. Contrary to parametric regression, nonparametric regressions assume that the relationship between the response (dependent) and the explanatory (independent) variables is not predetermined, with a non-explicit spectral band relationship, transformations, or fitting functions. They can further be split into linear or nonlinear regression models. Machine learning (ML) algorithms are examples of nonparametric nonlinear regression models. ML models are essentially empirical (Jacobs et al., 2022) and data-driven, although Tedeschi (2022) argued that algorithm developments could entail some mechanistic elements. Physically based models are applications of physical laws establishing cause-effect relationships. Model variables are inferred based on specific knowledge, such as knowledge-driven or mechanistic models. Those models are typically radiative transfer models, such as PROSAIL (Berger et al., 2018), and process-based grass models, such as BASic GRAssland model (BASGRA, Höglind et al., 2020). Complementary, physically based and nonparametric models can be combined in a hybrid approach. Hybrid models combine nonparametric nonlinear regression models’ flexibility and computing efficiency with the general qualities of physically based methods.

Using parametric based on vegetation indices (**VI**) is one of the most used and simplest methods to estimate forage mass (Verrelst et al., 2015). However, the accuracy of the estimation, based on these indices, depends strongly on the choice of the VI formula and the spectral bands selected (Dusseux et al., 2022), in addition to limitations due to saturation problems in high vegetation sites (Verrelst et al., 2015) and collinearity between spectral bands, which requires a selection of spectral bands or VI.

**Figure 2.**
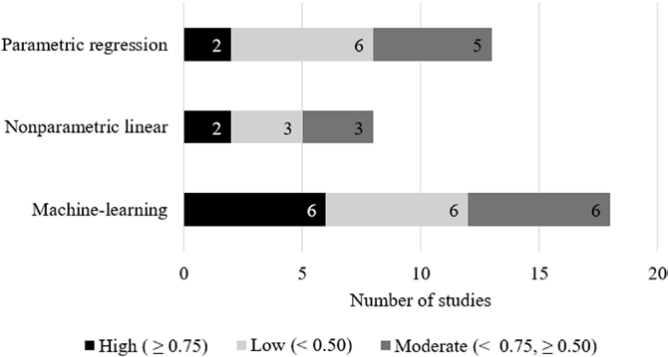
Number of studies that predicted grassland AGB based on satellite imagery data, classified by the precision (*R*^2^) of their model approach. Pearson’s chi-squared test, χ^2^ = 1.3375, *P* = 0.85 (performed in R software, version 4.2.2). Data from Table S1.

Firstly, we conducted a literature search on the Web of Science using keywords such as grassland, pasture, satellite, biomass, and forage. Then, we selected relevant published papers to estimate pasture parameters (e.g., AGB, height, nitrogen content, fiber content) using satellite data from multispectral optical sensors (Table S1). Those estimated parameters were selected to be potentially resourceful for further applications in pasture management at the farm or paddock level. The adoption of ML algorithms has sharply increased since 2020s (Table S1). In general, AGB is the main estimated parameter with the largest number of publications (Wang et al., 2022b), probably because of its relevance in terms of grassland production. Interestingly, the precision (*R*^2^) of models to estimate AGB (Table S1) was not associated with their category (parametric regression models, nonparametric linear models, ML models; Fig. 2), although the proportion of parametric regression models with high precision (*R*^2^ ≥ 0.75; 15%) tended to be lower than ML models (33%). A detailed discussion of the advantages and limitations of various models used to retrieve different grassland parameters is provided below.

### Parametric regression models

The simplest and the first approach used to explore satellite data on grasslands and rangelands was through linear regression models that related biophysical vegetation parameters and VI from optical sensors (Friedl et al., 1994; Todd et al., 1998; Ren and Feng, 2015; Verrelst et al., 2015). Eventually, the non-linearity between biophysical vegetation parameters and VI were fitted in nonlinear regression models (Verrelst et al., 2015), such as exponential (Edirisinghe et al., 2011), power (Jin et al., 2014), logarithmic (Jin et al., 2014), and others.

**Figure 3.**
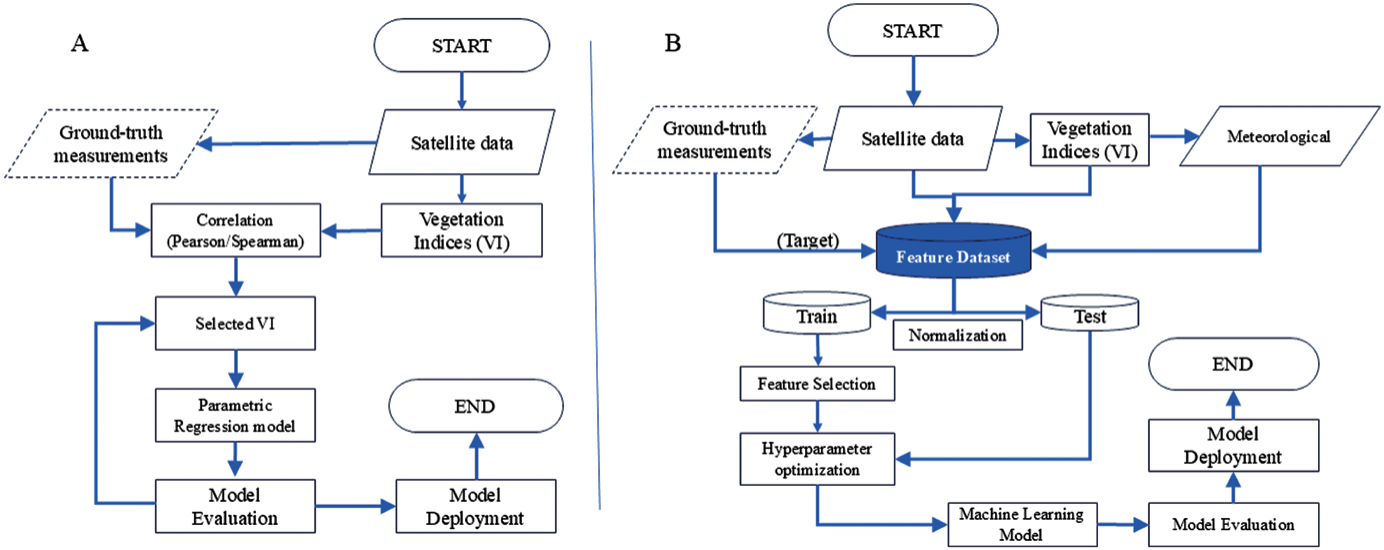
Flowchart of generalized modeling procedures used to describe the relationship between satellite imagery data and grassland parameters. (A) Parametric regression models, (B) supervised machine learning models (adapted from Fernandes et al., 2024).

Irrespective of being linear or nonlinear parametric regression models, building these models invariably requires the collection of field or ground-truth data on the target or vegetation variables. In general, only one VI is used as the independent variable in parametric regression models (Todd et al., 1998; Edirisinghe et al., 2011; Jin et al., 2014; Ren and Feng, 2015). A flowchart of generalized parametric regression procedures is illustrated in Fig. 3A. The fundamental simplicity of VI is their greatest advantage. The earliest implementations of broadband sensing satellites gave rise to VI-based approaches because of the limited amount of computer power available at the time and only a restricted number of spectral bands (Verrelst et al., 2015).

The most used VI in these models are those that relate the reflectance in the red (~ 690 nm) region of the spectrum, where chlorophylls in green leaves strongly absorb light, and in the NIR (~ 850 nm) region of the spectrum, where cell walls strongly scatter (reflect and transmit) light (Glenn et al., 2008). The most-known VI is the normalized difference vegetation index (**NDVI**): NDVI = (NIR—red)/(NIR + red), where NIR and RED are the reflectance values in their respective regions of the spectrum received at the sensors. Both NDVI and other related VI are functional variants of the simple ratio (**SR**) between NIR and RED (SR = NIR/ red). The NDVI normalizes values between −1 and +1; in which dense vegetation results in a high NDVI, while soil background shows low NDVI but positive, and water shows negative NDVI due to its strong absorption of NIR (Glenn et al., 2008).

A constant concern in constructing these models has been the interference of bare soil, especially in arid and rangeland regions, where green vegetation cover is low. Then, soil-adjusted vegetation indices have been frequently suggested as a proxy in regression models to estimate above-ground green biomass in low green vegetation cover areas (Ren and Feng, 2015; Jansen et al., 2018; Bretas et al., 2021). Nonetheless, Ren and Feng (2015) did not observe any improvements in soil-adjusted vegetation indices over the soil-unadjusted SR and NDVI as proxies in parametric linear regression models to predict above-ground green biomass in grasslands with low green vegetation cover (< 30%).

One of the issues observed when using parametric linear regression models to estimate AGB in grasslands with high forage accumulation rates, such as tropical forages (Bretas et al., 2021) or ungrazed rangelands (Todd et al., 1998), is the substantial interference of dry or senescent mass, which results in low predictability of these models. Indeed, Glenn et al. (2008) discussed that vegetation parameters are often only moderately correlated with satellite-derived VI or their derivatives, and remote sensing models that use VI to estimate these parameters are subject to error and uncertainty. Moreover, in areas with high green vegetation cover, vegetation indices, particularly the NDVI, approach a saturation level after a specific leaf area index or above-ground green biomass, so linear regressions might not be suitable and result in poor precision and accuracy.

When applying a single VI in regression equations to estimate grassland biomass, a major concern was related to the time and site-specificity of those models (Ren and Feng, 2015). For instance, the variable performance of NDVI in arid and semi-arid areas, where there is a high influence of soil background, might be attributed to differences in soil types and colors, whose spectral signature contrasts (Eisfelder et al., 2012; Ren and Feng, 2015).

The mismatch between the size of the sampling site and the spatial resolution of remotely sensed data is a common issue for biomass calculation based on satellite observation data (Eisfelder et al., 2012). Using MODIS, a low spatial resolution satellite, Ren and Feng (2015) overcame this problem by calculating VI from the selected pixel for each sampling site used to perform linear regressions, whose precision (*R*^2^) varied from 0.47 to 0.72.

### Nonparametric regression models

As previously discussed, parametric regression models require an explicit selection of spectral bands or VI. The main reason for that is the multicollinearity issue that can arise due to the high correlation between the reflectance of different regions of the spectrum, as well as between VI (Todd et al., 1998; Sibanda et al., 2016; Bretas et al., 2021), intrinsic to the characteristics of the electromagnetic spectrum. When 2 or more independent variables in a data frame strongly correlate with one another in a regression model, a statistical phenomenon known as multicollinearity happens. Multicollinearity would increase the standard error of the coefficients, making them unstable and unreliable, which will lead to a wrong conclusion on the model (Daoud, 2017). Nonetheless, the advancements in satellite sensors, which cover multiple wavebands or hyperspectral sensors, demand more flexible models that can combine different data structure features nonlinearly (Verrelst et al., 2015).

#### Nonparametric linear regression models.

 The simplicity and optimal performance of the nonparametric linear regression models are their main advantages (Verrelst et al., 2015). However, the use of more than one VI or selected spectral bands in multiple linear regressions (MLR) requires attention and caution. MLR recursively applies multiple regression several times by removing (stepwise) variables with the weakest correlation. An optimal variable set is obtained at the end of the recursive process (Verrelst et al., 2015). The MLR models in grazing satellite-driven data for grazing systems have been mainly applied to estimate total and green dry AGB of natural grasslands of Brazilian Pampa Biome (Guerini Filho et al., 2020), temperate grasslands (Ali et al., 2017; Wang et al., 2019) and tropical pastures (Bretas et al., 2021), with low (*R*^2^ < 0.50) to moderate precision (*R*^2^ < 0.75, Fig. 2).

Partial least squares (**PLS**) regression is a frequently applied technique in multivariate statistical analysis when independent variables are highly correlated (Li et al., 2002), so one of the most widespread and efficient techniques to analyze spectroscopy data (Sibanda et al., 2016; Askari et al., 2019; Fernández-Habas et al., 2021) and tackle their intrinsic collinearity problem (Verrelst et al., 2015). Its advantage over other algorithms is that few hyperparameters need to be set in PLS regression (**PLSR**); that is, only the number of latent variables (partial least square components) used to decompose the predictors and responses, which has been usually determined by cross-validation (Askari et al., 2019; Fernández-Habas et al., 2021).

Using the satellite Sentinel-2 to estimate dry AGB and crude protein in temperate grasslands, Askari et al. (2019) reported that PLSR and stepwise MLR models were sufficiently robust, although PLSR yielded a slightly better model than MLR. The accuracy of the models was reasonable for dry AGB and insufficient for crude protein estimation. The authors also highlighted the importance of the red-edge region of the spectrum on the predictability of dry AGB and the NIR range on the predictability of crude protein.

In another study, PLSR models were used to predict crude protein (**CP**), neutral detergent fiber (**NDF**), acid detergent fiber (**ADF**), and enzyme digestibility of organic matter in a temperate Mediterranean grassland using the satellite Sentinel-2 (Fernández-Habas et al., 2021). The authors observed that models to predict CP showed moderate predictive performance, while models to predict NDF showed poor predictive performance and models to predict ADF and enzyme digestibility of organic matter were not significant. The contribution of the spectrum’s red edge region and short-wave infrared (SWIR) region to estimate CP and NDF was also stressed.

#### Machine learning algorithms.

It is also known as nonparametric nonlinear regression models. The exponential evolution of digital computers has pushed forward a diversity of nonlinear nonparametric models, referred to as ML regression algorithms, that assume a non-explicit relationship between features and target variables. Machine learning methods require large data sets to better understand the patterns hidden inside the data. In remote-sensing-based data, a large set of features, including spectral bands and their VI or their diverse combinations, as well as meteorological and topographic data, can be used as independent predictive variables for modeling. Nonetheless, excessive features can harm theodel’s capacity to make predictions, make the model difficult to interpret for qualitative evaluation, and be computationally expensive (Dusseux et al., 2022; Fan et al., 2022).

Using feature selection, one can choose the best feature set from all the features to minimize the amount of processing power required, shrink the size of the feature space, and enhance the model’s readability and generalizability (Fan et al., 2022). Dusseux et al. (2022) suggested that feature selection methods could be divided into filter, wrapper, and intrinsic. Filter feature selection methods use statistical techniques to evaluate the relationship between each input feature and the target variable, ranking the most suitable features to be used in the model (e.g., Pearson’s correlation, Spearman’s correlation). By building many models with various subsets of input features, wrapper feature selection methods look for well-performing subsets of features and choose the features that provide the best-performing model based on a performance metric (e.g., recursive feature elimination, **RFE**). Certain ML techniques automate the process of feature selection during the model-learning phase. These methods could be called intrinsic feature selection strategies. This covers techniques like decision trees, including ensembles of decision trees like the random forest feature selection, and penalized regression models like Least Absolute Shrinkage and Selection Operator (**LASSO**), Ridge, and ElasticNet.

In general, the use of a feature selection algorithm improved the performance (precision and/or accuracy) of ML models (Fan et al., 2022; Wang et al., 2022; Zhang et al., 2023), but depended on the feature selection algorithm (Wang et al., 2022; Zhang et al., 2023). Modeling the grassland AGB using Sentinel-2 satellite images, Fan et al. (2022) used the RFE, based on random forest feature importance, and observed that the accuracy of the models gradually rose and eventually saturated as the number of features increased, reaching the highest accuracy with 5 input features in a set of total 27 features. The feature selection results indicate that features associated with temperature, altitude, humidity, and leaf water content significantly influence grassland forage mass.

Machine learning algorithms can be broadly categorized into supervised learning and unsupervised learning. Each category has specific algorithms that are particularly effective for processing and interpreting complex data derived from satellite images. Many ML algorithms have been used to analyze satellite imagery to estimate pasture forage mass and chemical composition, predominantly supervised learning (Table 2).

**Table 2.** Machine learning algorithms and their main applications in grazing systems based on satellite imagery

ML algorithms	Description	Examples of applications in grazing systems
Randon Forest (RF)	• An ensemble learning method that constructs multiple decision trees during training and outputs the mean prediction of individual trees. • Known for its robustness and ability to handle high-dimensional data002E	• Forage mass (Ramoelo et al., 2015; Wang et al., 2017; Wang et al., 2019; Meng et al., 2020; Reis et al., 2020; Bretas et al., 2021; Dusseux et al., 2022; Fan et al., 2022; Y. Wang et al., 2022; Bretas et al., 2023b; Fernandes et al., 2024) • Leaf area index (Wang et al., 2019) • Canopy height (Raab et al., 2020; Reis et al., 2020; Bretas et al., 2023b) • Nitrogen content (Ramoelo et al., 2015; Mashiane et al., 2023; X. Zhang et al., 2023) • Crude protein content (Raab et al., 2020; Fernandes et al., 2024) • Fiber (ADF^1^) content (Raab et al., 2020) • Fiber (NDF^2^) content (Fernandes et al., 2024) • Phosphorus content (X. Zhang et al., 2023) • Potassium content (X. Zhang et al., 2023)
Support Vector Regression (SVR)	• An extension of support vector machines (SVM), primarily used for regression problems. • Aim to find a function that approximates data points within a specified margin of tolerance while minimizing the prediction error. • Key advantages include the ability of handling high-dimensional data and robustness to overfitting, especially when dealing with small datasets	• Forage mass (Wang et al., 2019; Meng et al., 2020; Dusseux et al., 2022; Y. Wang et al., 2022; Vahidi et al., 2023; Fernandes et al., 2024) • Leaf area index (Wang et al., 2019) • Nitrogen content (X. Zhang et al., 2023) • Crude protein content (Fernandes et al., 2024) • Fiber (NDF^2^) content (Fernandes et al., 2024) • Phosphorus content (X. Zhang et al., 2023) • Potassium content (X. Zhang et al., 2023)
Gradient Boosting Regression Tree (GBRT)	• Another ensemble technique that builds trees sequentially, with each new tree correcting the errors of the previous ones. • Effective in improving prediction accuracy and handling complex datasets.	• Forage mass (Fan et al., 2022; Y. Wang et al., 2022)
eXtreme Gradient Boosting (XGBoost)	• An advanced implementation of gradient boosting that includes regularization to prevent overfitting. • Known for its speed and performance, making it suitable for large-scale satellite data analysis.	• Forage mass (Reis et al., 2020; Fan et al., 2022) • Canopy height (Reis et al., 2020)
Cubist	• A rule-based machine learning algorithm that combines decision trees with linear regression models. • Create a series of rules based on the input data and fits linear models to the data points that fall under each rule.	• Forage mass (Fan et al., 2022)
Artificial Neural Networks (ANN)	• Computational models inspired by the human brain, consisting of interconnected layers of neurons. • Particularly effective for complex pattern recognition tasks.	• Forage mass (Ali et al., 2017; Meng et al., 2020; Dusseux et al., 2022; Y. Wang et al., 2022; Vahidi et al., 2023) • Classification of mowing events (Komisarenko et al., 2022)
Convolutional Neural Networks (CNN)	• Type of deep learning algorithm primarily used for image recognition and classification. • Adept at handling the spatial hierarchies in images due to their convolutional layers that scan input images for features.	• Grassland land use intensity (Lange et al., 2022)
Adaptive-Neuro Fuzzy Inference Systems (ANFIS)	• Combine neural networks and fuzzy logic principles to model complex, nonlinear relationships. • Useful for handling the uncertainty and variability inherent in ecological data	• Forage mass (Ali et al., 2017)

^1^Acid detergent fiber.

^2^Neutral detergent fiber.

Supervised learning algorithms are trained using labeled data, which means that the input data (in this case, satellite images) is already associated with known output values (forage mass, protein, and fiber content). Fig. 3B illustrates a flowchart of generalized supervised machine learning modeling procedures.

One of the most widely ML algorithms used in forage mass estimation is the random forest (**RF**, Table 2) due to its advantages of processing high-dimensional vectors, reducing over-fitting, fast training speed, and noise immunity to a certain extent (Fan et al., 2022). The bootstrap aggregation approach of an ensemble learning algorithm includes the RF regression algorithm, which combines numerous random regression trees to improve regression accuracy. The training set’s data is randomly extracted by these regression trees, which then evenly vote to provide an average as the outcome. The random vectors that are sampled independently are used to build the parallel regression trees in the RF. By averaging the outcomes of each regression tree, the model’s result is obtained (Belgiu and Drăgu, 2016).

Support vector regression (**SVR**) is an extension of support vector machines (**SVM**), primarily used for regression problems. SVR aims to find a function that approximates data points within a specified tolerance margin while minimizing the prediction error. The key advantages of SVR include its ability to handle high-dimensional data and its robustness to overfitting, especially when dealing with small datasets (Mountrakis et al., 2011; Fernandes et al., 2024).

Meng et al. (2020) applied RF and SVR algorithms to predict forage mass in alpine grasslands on the Tibetan Plateau, China. Their study observed that the RF model provided the highest accuracy, attributed to its ability to handle complex interactions between predictors and its resistance to overfitting. Conversely, Fernandes et al. (2024) observed that SVR slightly outperformed RF model in predicting leaf forage mass of tropical forages during the growing season.

Gradient Boosting Regression Tree (**GBRT**) is another ensemble learning model. This approach creates an upgraded estimation model with gradients by combining numerous regression trees. There is a string of these regression trees. The GBRT is intended to compute the residuals between the fitted and true values after fitting the data using the first regression tree. The second regression tree then keeps fitting the residuals from the previous step to lower the residuals between the total fitted and true values. The quantity of regression trees determines the number of iterations (Fan et al., 2022). A multiple regression tree ensemble learning approach, eXtreme Gradient Boosting (**XGBoost**) is an extension of GBRT. Whereas GBRT makes use of first-order derivatives, XGBoost presents a second-order Taylor formula. Furthermore, XGBoost could build a new tree based on the old tree and adjust the residuals. Cubist is a rule-based regression tree algorithm and an extension of the M5 model tree. The algorithm generates rule-based models with one or more rules, each rule containing a set of criteria associated with a multivariate linear sub-model. Each linear model is a “leaf” of Cubist. Therefore, the Cubist model is efficient and easy to understand (Fan et al., 2022). Fan et al. (2022) observed that RF models outperformed cubist, GBRT, and XGBoost models for estimating the grassland AGB of the Tibetan Plateau during the growing season, using Sentinel-2 images in tandem with meteorological and topographic data.

The estimations of nitrogen (or CP) content have the potential to provide insight into animal feeding patterns and distribution. Satellite remote sensing sensors with red-edge bands in tandem with ML algorithms have boosted the accuracy of mapping vegetation biochemical concentrations because this spectrum is the maximum slope point in healthy vegetation. Mashiane et al. (2023) focused on the use of the RF regression algorithm to predict nitrogen content in grass species. This study used RF to analyze a combination of spectral bands and vegetation indices derived from Sentinel-2 multispectral imagery. The study found that the combined model, which included both spectral bands and vegetation indices, performed the best with an accuracy of 85%, compared to 80% using only bands and 81% using only vegetation indices. Fernandes et al. (2024) employed SVR and RF machine learning algorithms to assess these algorithms’ performance in predicting forage mass, crude protein, and fiber content of tropical pastures using Sentinel-2 imagery combined with meteorological data. The findings indicated that SVR models generally outperformed RF models in this context. Specifically, the SVR models achieved higher precision and accuracy, with R² values reaching as high as 0.66 for crude protein and 0.57 for fiber content estimation. The better performance of SVR in this study highlights its strength in dealing with small to moderate datasets and its capability to manage high-dimensional space more efficiently than RF, which is crucial when incorporating diverse data types like meteorological inputs and spectral indices. In another study, Raab et al. (2020) utilized the RF regression technique to integrate Sentinel-2 optical data and Sentinel-1 radar data for predicting rangeland quality indicators like ADF and CP. The incorporation of Sentinel-1 radar data, which provides information about the physical structure of the vegetation, significantly enhanced the predictive accuracy of the RF models. The study reported strong R² values of 0.79 for ADF and 0.72 for CP predictions. This study showcases the effectiveness of RF in processing high-dimensional and mixed-type data, including both spectral and radar inputs, to yield high accuracy in ecological predictions.

Artificial Neural Networks (**ANN**) are computational models inspired by the human brain, consisting of interconnected layers of neurons. They are particularly effective for complex pattern recognition tasks. In the context of satellite imagery, ANN has been used to analyze spectral data to predict vegetation biomass. Convolutional Neural Networks (**CNN**) are a type of neural network classified as a deep learning algorithm that is specifically designed for processing structured grid data like images. CNN are adept at handling the spatial hierarchies in images due to their convolutional layers that scan input images for features. Adaptive neuro-fuzzy inference systems (**ANFIS**) combine neural networks and fuzzy logic principles to model complex, nonlinear relationships. ANFIS is particularly useful for handling uncertainty and variability inherent in ecological data (Ali et al., 2017). They (Ali et al., 2017) observed that ANN and ANFIS outperformed MLR in estimating biomass in intensively managed grasslands in Ireland. The models tended to underestimate higher biomass values, possibly due to the saturation of VI estimated from 2 spectral bands (red and NIR) derived from MODIS satellite data. Variability in data from a site with more intense grazing practices led to lower accuracy than other sites with less intense grazing practices. Komisarenko et al. (2022) used ANN to predict grassland mowing events during the vegetative season in Estonia, using Sentinel-1 and Sentinel-2 images. The proposed model significantly outperforms the state-of-the-art technique and achieves event accuracy of 73.3% and end-of-season accuracy of 94.8%. Using Sentinel-2 time series images and CNN algorithms, Lange et al. (2022) developed a methodology to estimate grassland land use intensity (**LUI**) based on mapping and classification of grazing intensity, mowing frequency, and fertilizer application of areas across Germany. The authors reported an overall classification accuracy of up to 66% for grazing intensity, 68% for mowing, 85% for fertilization, and an *R*^2^ of 0.82 for subsequently depicting LUI.

The choice of algorithm typically depends on the nature of the data, the specific requirements of the task, and the availability of labeled datasets. In satellite imagery for estimating pasture forage, a combination of these algorithms can be used to leverage the labeled data from field samples and the broader patterns visible across larger, unlabeled satellite data sets. This integrated approach can lead to more accurate and robust models for predicting pasture health and composition.

### Physically based models

Remote sensing data can also be integrated into mechanistic and dynamic models to enhance their performance in predicting grassland parameters, for instance, in the inversion of radiative transfer models (**RTM**) (Quan et al., 2017; Punalekar et al., 2018; Schwieder et al., 2020; Dehghan-Shoar et al., 2023) and process-based grass models (**PBGM,**Huang et al., 2021). RTM are based on the physical principles of radiative transfer theory, which describes how light is absorbed, transmitted, and scattered within plant canopies (Berger et al., 2018). It considers factors like leaf optical properties, canopy structure, and the dynamics of radiation absorption, reflection, and transmission within the vegetation canopy. The model typically includes parameters like leaf area index (**LAI**), chlorophyll content, and other canopy characteristics that influence how the vegetation responds to different wavelengths of light (Berger et al., 2018). The RTM combines with satellite-observed radiance or reflectance through inversion, which adjusts RTM parameters to match observed reflectance values (Verrelst et al., 2015; Berger et al., 2018). However, this process is often under-determined and ill-posed, meaning multiple parameter combinations can produce the same reflectance, leading to ambiguity.to important metrics (Verrelst et al., 2015; Zhang et al., 2023a), which could be a limitation in practical applications (Zhang et al., 2023a).

Different RTM models have been applied in grassland such as PROSAIL (Quan et al., 2017; Punalekar et al., 2018; Zhang et al., 2023a), Soil-Leaf-Canopy (SLC, (Schwieder et al., 2020), and Soil-Plant-Atmosphere Radiative Transfer (SPART, (Dehghan-Shoar et al., 2023). Optical satellite data and RTM inversion techniques have been used to retrieve grassland biomass (Quan et al., 2017; Punalekar et al., 2018; Zhang et al., 2023a), LAI (Quan et al., 2017; Punalekar et al., 2018) and N content (Dehghan-Shoar et al., 2023). Aimed to estimate AGB in a plateau grassland in China, (Quan et al., 2017) observed that RTM-based inversion method (PROSAIL) showed higher accuracy (*R*^2^ = 0.64 and RMSE = 42.67 g m^−2^) than the exponential regression (*R*^2^ = 0.48 and RMSE = 41.65 g m^−2^) and the ANN (*R*^2^ = 0.43 and RMSE = 46.26 g m^−2^), but worse performance than PLSR (*R*^2^ = 0.55, RMSE = 37.79 g m^−2^).

The PBGM is a mechanistic model developed to simulate grass growth processes under different environmental conditions and field scales (Huang et al., 2021) to assist in optimal practices for grassland management. Typically, PBGM uses a set of parameters to describe the empirical relationship between state variables and fluxes, which require calibration to produce the best performance and greater predictive accuracy for different grasses and forages. Although real observations (field records) are the most common data source, satellite-derived data have been used successfully to calibrate those models, mainly in areas with very few or even no observations in targeted study areas (Huang et al., 2021).

Popular data assimilation methods, including Bayesian calibration and the updating method ensemble Kalman filter, were applied to assimilate satellite-based MODIS-derived information (LAI, gross primary production, and evapotranspiration) into the BASic GRAssland model (BASGRA_N;(Höglind et al., 2020)).

These models differ in spatial complexity, input requirements, and the computational power needed. The choice of model depends on the specific application, such as biomass estimation or nitrogen, and the level of heterogeneity in the grassland being studied. Optimizing the model parameter specific for grass species and cultivars should be targeted before updating model state variables (Huang et al., 2021). Although parameters that were optimal to a particular site were not necessarily transferable to other sites, physically based models may offer the advantage of estimating grassland parameters at a large scale without the need to collect field measurements (ground-truth data; Quan et al., 2017; Punalekar et al., 2018; Zhang et al., 2023a).

## Practical Applications of Satellite-Based Vegetation

The needs of farm operators, the type of grass, the soil, and the climate influence how grasslands are managed over time and space (Ogungbuyi et al., 2023). Moreover, grasslands are widely distributed in various conditions and are pretty diverse (high or low, slope or flat, dry or wet, natural or planted (Reinermann et al., 2020; Ogungbuyi et al., 2023). Therefore, high spatial, temporal, and spectral resolution remote sensing data are needed to implement a timely pasture biomass estimation model and develop an innovative and operational management decision-support system to characterize and study them at different scales, from local to regional.

Satellite-based vegetation data offers significant potential for improving grazing management by providing large-scale, frequent, and objective assessments of pasture conditions, forage availability, and environmental impacts (Wardropper et al., 2021). Moreover, satellite images collected over time can be analyzed to observe long-term trends and changes in grazing areas (Ali et al., 2017; Beutel et al., 2019; Jones et al., 2021). These historical insights are resourceful in understanding the impacts of different grazing practices and can guide the implementation of more sustainable methods. While challenges exist, ongoing advancements in technology and analytics promise to enhance the application of satellite data in grazing management.

### Integrating the animal component in satellite-based vegetation models

Previous models discussed in this review provided the foundation for estimating grassland parameters based on satellite remote sensing. Nevertheless, the animal-grassland relationship is critical to the productivity and sustainability of grazing systems. Adjusting stocking rates and predicting average daily gain (**ADG**) are critical aspects of practical application in precision grazing management (Bretas et al., 2023). Thus, satellite-based vegetation data can significantly enhance decision-making by providing timely and accurate information on pasture conditions and forage quality. Stocking rate, the number of animals per unit area for a specific period, is a pivotal factor in grazing management (Bretas et al., 2023). Overstocking can lead to overgrazing, reduced pasture productivity, and environmental degradation, while understocking may result in underutilized resources and economic inefficiencies (Aiken, 2016). Satellite data, particularly forage mass estimates, can be used to monitor forage availability in real-time. This allows grazing managers to adjust stocking rates dynamically, matching livestock numbers with forage supply to optimize grazing pressure and maintain pasture health (Wardropper et al., 2021).

The nutritive value and quantity of available forage directly influence livestock productivity. Satellite models that provide data on biomass and chemical composition (such as CP, and fiber content) can help predict potential gain by estimating the nutritive value of the forage. Moreover, integrating satellite-derived forage mass and nutritive value data with mechanistic nutritional models can yield predictive models for ADG. This integration may enable producers to make informed decisions about feed supplements, grazing rotations, and overall herd management strategies to maximize growth rates and economic returns. Some studies highlight the potential implementation of satellite data for adjusting stocking rates and predicting ADG (Phillips et al., 2009; Jansen et al., 2021; Pearson et al., 2021; Kearney et al., 2022).

Phillips et al. (2009) conducted one of the first studies to develop a method for estimating short-term grazing capacity on small paddocks by integrating geospatial data and cattle nutrition information. Utilizing GIS, the study combined high-resolution satellite imagery (from Landsat and ASTER satellites) and field data to assess forage quality and quantity, thereby determining the number of days specific paddocks can meet the nutritional needs of beef cattle. Although evaluated in a controlled trial involving actual cattle grazing, the model highlighted discrepancies between estimated and actual grazing days, in which satellite-based models underestimated grazing capacity by 4 d while the field-based model overestimated grazing capacity by 1 d, suggesting further refinement. However, the authors concluded that integrating cattle nutrition and forage data in GIS can assist in adjusting stocking rates and enhance precision in grazing management.

Another study (Jansen et al., 2021) aimed to improve grazing management by integrating satellite-based vegetation data and conventional monitoring indicators across grasslands in northeast Oregon. The authors employed a combination of satellite imagery analysis using Landsat data and field surveys to measure vegetation biomass and grazing impacts. Biomass metrics derived from satellite data were compared against on-the-ground indicators of grazing pressure, such as stocking rate and field-based utilization. The main results indicated that specific remotely sensed biomass metrics, notably the mean biomass in the fall and relative difference in biomass between summer and fall, had the most consistent and significant correlations with grazing indicators. These relationships were used to create visual maps to aid adaptive management decisions. In conclusion, the study demonstrates the utility of satellite-based vegetation data in rangeland management, showing how these methods can be aligned with traditional monitoring to provide a more comprehensive understanding of grazing impacts. This approach aids in making more informed, data-driven decisions for grazing management, potentially enhancing ecological and economic sustainability.

The relationship between satellite-derived VI and the live weight changes of beef cattle under extensive grazing conditions was explored by Pearson et al. (2021). The authors utilized Sentinel-2 satellite imagery to calculate VI and walk-over-weighing (WoW) stations to monitor cattle weight, and the authors focused on integrating remote sensing technology and animal biometrics. Over 2 yr, data analysis involved linear mixed-effects regression models and random forest machine learning to explore correlations between VIs and livestock weight, incorporating variables like rainfall and time. The results confirmed a significant positive relationship between all VI studied and live weight changes, demonstrating the predictive power of combining VI with meteorological data. Machine learning models corroborated these findings, achieving moderate accuracy in predicting live weight changes based on environmental and temporal data. These findings suggest that remote monitoring can significantly enhance the management of pasture availability and livestock weight, potentially leading to improved grazing practices and productivity in extensive cattle farming operations.

Kearney et al. (2022) predicted spatial-temporal patterns of diet quality and significant herbivore performance using a combination of field data and satellite-derived phenological metrics. The study utilized a robust modeling approach that combined pseudo-daily satellite time-series data with multi-temporal field observations of forage conditions to predict diet quality and the subsequent body weight gain of free-ranging yearling cattle in a shortgrass steppe throughout the grazing season, from mid-May to October. The analysis found strong relationships between field-measured diet quality and satellite-derived phenological metrics, particularly those related to the timing and rate of green-up and senescence. The study confirms that the diet quality estimates derived from these satellite metrics can predict monthly BW gains with a coefficient of determination (*R*^2^) of 0.68 across a range of herbaceous production scenarios. Nonetheless, the models demonstrated better temporal than spatial performance, indicating a need for accurate vegetation maps and robust field data collection to improve predictive accuracy across both dimensions.

Yet, regarding beef cattle production, cow-calf production is predominantly based on grazing systems as the first sector of the supply chain (Asem-Hiablie et al., 2015). Therefore, developing models to select for improved beef cow efficiency is pivotal to improving competitiveness and profitability (Tedeschi et al., 2006) and ensuring the sustainability of the entire beef supply chain. For instance, Tedeschi et al. (2006) developed a mathematical model to compute a cow energy efficiency index based on the diet of the cow’s metabolizable energy required for a given calf weaning weight. The model embodied a nutritional model that computes the energy requirements of individual beef cows and their calves and computes the energy balances for the herd for each day of the year to evaluate the balance between herd numbers and requirements with the forage available. Based on Tedeschi’s model (Tedeschi et al., 2006), Adams et al. (2023) prototyped a conceptual hybrid model combining an existing mechanistic nutrition model with pasture forage mass estimated using time series satellite images. 1n the satellite-based grazing cow-calf model, the Ruminant Nutrition System (**RNS**) was used to estimate animal nutrient requirements (Tedeschi and Fox, 2020), where potential dry matter intake (**DMI**) was estimated based on herd size for each animal category. Forage allowance was estimated by dividing total forage mass (estimated by satellite imagery) by total herd live weight. Average weaning weight was used to predict cow milk yield and total metabolizable energy requirements, which was used to estimate the required DMI (**DMR**). Actual forage allowance was correlated to DMR, and it simulated the forage allowance needed to support increased DMI and, consequently, increased milk yield to achieve a targeted average weaning weight. Although the model is still in development, the authors concluded that it provides a fundamental and resourceful framework for a decision-support tool for producers to optimize their cow-calf operations while producing ideal weaned calves.

In the context of PLF, an optimal stocking rate might be adjusted by combining virtual fencing and satellite-based vegetation data to leverage pasture and rangeland management. In recent years, virtual fencing has emerged as a promising technology to control livestock movement without traditional physical barriers (Goliński et al., 2023). By utilizing GPS-enabled collars and real-time data, livestock can be guided across grazing lands, offering a great opportunity to optimize pasture use and prevent overgrazing (Stevens et al., 2021; Menendez et al., 2022; Heins et al., 2023). This combination would allow for a dynamic, data-driven approach to grazing management, where real-time satellite data on forage biomass and quality informs virtual fencing adjustments. Despite being very promising, studies evaluating the performance and effectiveness of combining virtual fencing and satellite-based (or remote sensing) grassland data are still incipient (Hamidi et al., 2023; Fuchs et al., 2024; Parsons et al., 2024). (Hamidi et al., 2023) explored and confirmed the potential of monitoring animals using virtual fencing collars, integrating this approach with UAV data to enable continuous tracking of both animals and pasture. The study concluded that combining behavioral analysis with UAV-based spatial assessments of pasture usage, alongside the fencing capabilities of the VF system, offers a sustainable, fine-scale decision-support tool for grassland management based on a polygon grid system, known as ‘grid grazing.’ Though Hamid’s work utilized UAV remote sensing rather than satellite remote sensing (the focus of this review), it demonstrated the promising potential of combining technologies to enhance grassland management and support decision-making in livestock operations.

### Web services and platforms for monitoring, management, and planning of grasslands

Diverse web platforms based on satellite remote sensing models are available to land managers to assist them in grassland monitoring, management, and planning (Table 3). Certainly, these online platforms can transfer knowledge to technology, enabling the deployment of precision technologies to support farms in grazing management (Wardropper et al., 2021; Heins et al., 2022; Bretas et al., 2023). Some are freely accessible, while others require an investment for commercial use. The Australian “Pastures from Space” project (https://agric.wa.gov.au/n/7700) was one of the pioneers to provide grassland information (biomass and growth rate) using moderate to high-resolution satellites for remote sensing (Chizzotti et al., 2022).

**Table 3.** Non-exhaustive list of web platforms that offer services on grazing systems based on satellite derived models

Service name	Location (origin)	URL	Reference
*Free services*			
FORAGE	Australia	https://www.longpaddock.qld.gov.au/	(Zhang and Carter, 2018)
RangeSAT	Oregon/Idaho, USA	https://www.rangesat.org/	(Jansen et al., 2018)
Rangeland Analysis Platform (RAP)	Western USA	https://rangelands.app/	(Jones et al., 2021)
GrassCast	Central Plains/Southwest, USA	https://grasscast.unl.edu/	(Hartman et al., 2020)
*Commercial services*			
Pasture.io^1^	Australia	https://pasture.io/	
Cibolabs^2^	Australia	https://www.cibolabs.com.au/	
SPACE^3^	New Zealand	https://www.lic.co.nz/products-and-services/space/	
Sigfarm^4^	Brazil/USA	https://www.sigfarmintelligence.com/	
Enriched^5^	USA	https://enriched.ag/	

^1^Uses satellite data to automate grazing management, offering real-time information on pasture growth, stocking rates, and grazing rotations.

^2^Specializes in offering satellite-based insights for livestock and rangeland management. It provides high-resolution data on vegetation biomass, soil moisture, and land cover changes to help optimize grazing strategies and monitor land health.

^3^Integrates satellite imagery and pasture growth modelling to deliver pasture cover data and analysis for farms.

^4^A remote sensing platform that helps monitor crop and pasture conditions, offering predictive analytics for yield and environmental monitoring.

^5^Focuses on providing satellite-based data for carbon farming, helping landholders improve soil health, sequester carbon, and monitor pasture performance.

Wardropper et al. (2021) compiled some freely available web-based climate services designed to aid ranchers and pastoralists in their decision-making process, and some use satellite data to estimate pasture parameters, mainly forage mass and forage growth. FORAGE (https://www.longpaddock.qld.gov.au/; Zhang and Carter, 2018) is a Queensland government platform that provides detailed reports on land conditions, using satellite data to monitor factors such as pasture growth, ground cover, soil erosion, and drought conditions. It offers a variety of reports tailored to landholders, focusing on aspects like long-term carrying capacity, pasture productivity, rainfall, and drought risk. RangeSAT (https://www.rangesat.org/; Jansen et al., 2018) is another valuable platform in the suite of remote sensing tools for monitoring rangelands and pasture systems. Developed by Montana State University, RangeSAT is designed to help ranchers, land managers, and conservationists make data-driven decisions about grazing and land management by providing regular satellite-based updates on vegetation productivity and condition. Rangeland Analysis Platform (RAP, https://rangelands.app/; Jones et al., 2021) is a platform designed specifically for rangelands and provides land managers with satellite-derived maps to monitor trends in vegetation, including bare ground, herbaceous cover, and shrubs, over time. GrassCast (https://grasscast.unl.edu/; Hartman et al., 2020) developed by the USDA and partners, utilizes satellite imagery to forecast grassland productivity across the Great Plains and Southwest regions of the United States. The platform integrates nearly 40 yr of historical data on weather and vegetation growth with seasonal precipitation forecasts to predict whether rangelands in individual grid cells (approximately 10 km × 10 km) will likely produce above-normal, near-normal, or below-normal amounts of vegetation. Similarly to freely available web-based platforms, commercial platforms provide services on grassland vegetation, but they provide additional tailored reports with customized insights into grazing management. Table 3 provides some examples of available commercial platforms.

Most platforms currently provide information centered solely on quantitative aspects of pasture, such as the available forage mass. In the future, however, these platforms could integrate predictive models for pasture nutritional value, including chemical composition like nitrogen or fiber content (Askari et al., 2019; Chabalala et al., 2020; Raab et al., 2020; Fernández-Habas et al., 2021; Pereira et al., 2022; Zhang et al., 2023; Fernandes et al., 2024). Combined with animal-specific data, information on pasture nutritional quality will advance precision nutrition models for grazing animals to a new level of insight (Tedeschi et al., 2019).

## Limitations, Challenges, and Future Perspectives

Satellite remote sensing offers significant advantages for pasture and rangeland management but also has notable challenges and limitations. In a more comprehensive view, we can categorize the challenges and limitations of satellite remote sensing into 3 types: 1) instrumentation: issues related to the data acquisition; 2) modeling: issues related to statistical methods to interpret and apply extracted data; and 3) usability: issues related to end-users adoption.

One key challenge in instrumentation, frequently highlighted in reviews (Wachendorf et al., 2017; Chizzotti et al., 2022; Bretas et al., 2023; Pinna et al., 2024), involves cloud cover, particularly limiting in optical sensors and mostly problematic in tropical or temperate regions where frequent cloudiness can hinder the acquisition of consistent data, leading to gaps in monitoring. A potential solution to address cloud cover interference involves fusing data from various satellites or alternative remote sensing platforms, such as UAV. However, this approach may increase computational costs and does not always result in improved accuracy (Raab et al., 2020). Another challenge could be spatial resolution because many free or accessible satellites like MODIS and Landsat provide data at coarse resolutions, which may not capture fine-scale variations in terms of paddock level. Additionally, temporal resolution can be a constraint, as the revisit time of satellites may not align with the rapid changes in vegetation due to grazing, rainfall, or seasonal shifts. Spectral limitations are also present, as some satellite sensors lack the specific bands necessary to capture detailed information on vegetation types or soil moisture, which is critical for accurate pasture assessment. Fortunately, the Sentinel-2, which was launched in 2015 by the European Space Agency, fulfills most of the spatial, temporal, and spectral resolution concerns because of its 13 spectral bands (from 443 to 2,190 nm) at high spatial resolution (from 10 to 60 m) with a revisit time of 5 d. Moreover, commercially available platforms such as Planet Scope and Worldview offer daily revisit times with great spatial resolution (1.8 m for Worldview and 3 m for Planet Scope), even though their number of multispectral bands is less than that of Landsat and Sentinel platforms (Pinna et al., 2024).

Regarding modeling, one key challenge is that ground truthing remains essential because satellite data often needs to be validated with on-the-ground measurements to ensure accuracy, which can be resource-intensive (Reinermann et al., 2020). Furthermore, data interpretation can be complex, requiring advanced expertise in image processing and statistical techniques, which can be a barrier for some users. So, several challenges and limitations arise when considering the models used in satellite remote sensing for pasture and rangeland management. One significant challenge is model accuracy—many models depend heavily on the quality of input data and assumptions about vegetation dynamics, which may not always reflect the complexity of rangeland ecosystems. For instance, models predicting forage biomass or nutrient composition frequently depend on the correlation between satellite-derived vegetation indices and ground-based measurements. However, the accuracy of these models can vary with the data acquisition frequency. Bretas et al. (2023) found that models developed with a 1-d gap between image capture and field sampling outperformed those with a 5-d gap, suggesting that finer temporal resolution positively influenced model accuracy. Similarly, Correa-Luna et al. (2024) emphasized that biomass data collected at least twice monthly (fortnightly intervals) is the minimum required to train ML models effectively and significantly reduce prediction error, underscoring the importance of finer temporal resolution.

Another limitation is the generalizability of models. Models developed for specific regions or ecosystems may not perform well when applied to different geographical areas or vegetation types without significant recalibration (Smith et al., 2023). This issue relies on data availability, where high-quality ground data needed for training or validating models is often scarce, particularly in remote or under-researched areas. Additionally, model overfitting is another concern; when models are trained on limited or particular datasets, they may perform well in those conditions but fail to predict accurately in broader or more variable conditions, reducing their robustness.

Moreover, addressing the uncertainty and bias in remote sensing models is crucial. Future work should emphasize refining machine learning algorithms to better account for environmental variability and reduce overfitting issues. A move toward more transparent and interpretable models would also allow for broader adoption among land managers, ensuring that the models are both scientifically robust and practically applicable.

Regarding usability, end-user adoption of satellite-derived models for grazing management hinges on several factors, including ease of use, data accessibility, cost-effectiveness, and perceived reliability. Wardropper et al. (2021) discussed a gap in research on socio-economic implications and adoption barriers of remote sensing technologies in pasture management. Farmers and land managers often face technological and financial barriers, which could be mitigated by developing user-friendly platforms that simplify data interpretation. Training or support services can also enhance users’ ability to integrate satellite-derived insights into their decision-making processes (Wardropper et al., 2021). Additionally, establishing trust in the model’s accuracy and relevance to specific grazing conditions is crucial. Understanding how to make these technologies more accessible and affordable for ranchers, especially in developing regions, could bridge the gap between scientific advancement and practical application, ensuring that remote sensing tools reach their full potential in sustainable rangeland management.

In the future, end-user experience with service platforms employing remote sensing for pasture management could be enhanced by integrating large language models (LLM). These models would enable interactive support, answer user questions, and offer pasture management insights customized to their specific farm conditions.

## Supplementary Material

skaf137_suppl_Supplementary_Material

## Abbreviations

ADF: acid detergent fiber
AGB: above-ground biomass
ANFIS: adaptive neuro-fuzzy inference systems
ANN: artificial neural networks
BOA: bottom-of-atmosphere
CNN: convolutional neural networks
CP: crude protein
DMI: dry matter intake
DMR: required DMI
GBRT: gradient boosting regression tree
GIS: geographic information systems
LAI: leaf area index
LASSO: least absolute shrinkage and selection operator
LIDAR: light detection and ranging
LUI: land use intensity
ML: Machine learning
MLR: multiple linear regressions
MM: mathematical models
NDF: neutral detergent fiber
NDVI: normalized difference vegetation index
NIR: near infrared
PBGM: process-based grass models
PLF: precision livestock farming
PLS: partial least squares
PLSR: PLS regression
RADAR: radio detection and ranging
RF: random forest
RFE: recursive feature elimination
RGB: red, green, and blue spectrum
RNS: Ruminant Nutrition System
RTM: radiation transfer models
SR: simple ratio
SVM: support vector machine
SVR: support vector regression
SWIR: short-wave infrared
TOA: top-of-atmosphere
VI: vegetation indices
UAV: unperson aerial vehicles
WoW: walk-over-weighing
XGBoost: eXtreme gradient boosting
