# Supplementary material for: ASAS-NANP symposium: mathematical modeling in animal nutrition: application of modeling innovations to support satellite remote sensing for sustainable grazing cattle management
Source: J Anim Sci. 2025 May 4;103:skaf137. doi: 10.1093/jas/skaf137 (PMC12351259; doi:10.1093/jas/skaf137)
Supplement: skaf137_suppl_Supplementary_Material [file skaf137_suppl_supplementary_material.docx]

Table S1. Detailed information of the studies used in this review.

| Source | Region | Sensor^1^ | Type of grass | Model^2^ | Target Variable^3^ | Input  Variable^4^ | R^2^ | RMSEP (%) |
| --- | --- | --- | --- | --- | --- | --- | --- | --- |
| ***Parametric regression models*** | | | |  |  |  |  |  |
| (Friedl et al., 1994) | Kansas, USA | Landsat | Tallgrass prairie | Linear | Dry AGB | SAVI | 0.45 – 0.54 |  |
| (Todd et al., 1998) | Colorado, USA | Landsat-5 | Shortgrass steppe | Linear | Dry AGB  (grazed) | NDVI  GVI  WI  RED | 0.66  0.67  0.62  0.64 |  |
|  |  |  |  |  | Dry AGB  (ungrazed) | RED | 0.35 |  |
| (Edirisinghe et al., 2011) | Southwest and Western, Australia | Landsat SPOT | Mediterranean pasture | Exponential | Dry green biomass | NDVI | 0.82 | 14% |
| (Ferreira et al., 2013)5 | Araguaia, Brazil | Landsat/ MODIS | Cerrado, *Brachiaria spp.* | Linear | Dry green biomass  % green cover | NDVI  NDVI | 0.46  0.92 |  |
| (Jin et al., 2014) | Xilingol, China | MODIS | Meadow steppe | Power  Logarithm  Linear  Linear  Linear | Fresh AGB | NDVI  DVI  EVI2  SAVI  MSAVI | 0.60  0.46  0.51  0.52  0.51 |  |
|  |  |  | Typical steppe | Power  Linear  Linear  Linear  Linear | Fresh AGB | NDVI  DVI  EVI2  SAVI  MSAVI | 0.57  0.39  0.51  0.52  0.50 |  |
|  |  |  | Desert steppe | Linear  Linear  Linear  Linear  Linear | Fresh AGB | NDVI  DVI  EVI2  SAVI  MSAVI | 0.48  0.47  0.49  0.49  0.49 |  |
| (Schucknecht et al., 2015) | Nigeria | SPOT | Sahelian savannah | Linear | Biomass | CFAPAR | 0.31- 0.49 |  |
| (Ren and Feng, 2015) | Inner Mongolia, China | MODIS | Temperate desert steppe | Linear | Fresh AGB | SR  NDVI  SAVI  MSAVI  OSAVI  TSAVI  ATSAVI  PVI | 0.72  0.71  0.61  0.56  0.67  0.58  0.56  0.47 | 29.7  30.5  35.4  37.6  31.8  36.6  38.1  41.4 |
| (Jansen et al., 2018)6 | Zumwalt Prairie, Oregon, USA | Landsat-7/8 | Temperate and warm-season grasses | Linear | Dry AGB  % green cover | NDTI  NDTI | 0.76  0.70 | 26.1  20.7 |
| (Meng et al., 2020) | Tibetan Plateau, China | MODIS | Alpine meadow | Exponential | Dry AGB | EVI  NDVI | 0.36  0.35 |  |
| (Bretas et al., 2021) | Brazil | Sentinel 2  Landsat 8 | *Brachiaria sp.* | Linear | Fresh AGB  Dry AGB | NDVI  EVI  OSAVI  NDVI | 0.73  0.72  0.73  0.25 |  |
| ***Nonparametric linear regression models*** | | | |  |  |  |  |  |
| (Ali et al., 2017) | Moorepark and Grange, Ireland | MODIS | Temperate grasslands | MLR | Dry AGB | VI, Red, NIR | 0.29 - 0.38 |  |
| (Wang et al., 2019) | Oklahoma, USA | Sentinel 2  Landsat 8 | Native grasslands | MLR | Dry AGB | NDVI, EVI, LSWI, | 0.69 |  |
| (Askari et al., 2019) | Moorepark, Ireland | Sentinel-2 | Temperate grasslands | MLR (stepwise) | Dry AGB  CP | Bands, VI | 0.81  0.58 |  |
|  |  |  |  | PLSR | Dry AGB  CP | Bands, VI | 0.82  0.62 |  |
| (Guerini Filho et al., 2020) | Santa Maria, Brazil | Sentinel-2 | Natural grasslands of Brazilian Pampa Biome | MLR | Green AGB  AGB | Bands, VI | 0.40 - 0.65  0.42 - 0.61 |  |
| (Bretas et al., 2021) | Brazil | Sentinel 2  Landsat 8 | *Brachiaria sp.* | MLR | Fresh AGB  Dry AGB | NDVI, EVI, OSAVI, Meteorological | 0.76  0.19 |  |
| (Fernández-Habas et al., 2021) | Andalusia, Spain | Sentinel-2 | Mediterranean grasslands | PLSR | CP  NDF | Bands, VI | 0.52  0.53 |  |
| (Alvarez-Mendoza et al., 2022) | Colombia | Sentinel-2 | Tropical pastures *Brachiaria sp.* | MLR | Dry AGB | VI | 0.63 |  |
| (Wang et al., 2022) | Qinghai Province, China | MODIS | Alpine meadows and grasslands | PLS | Dry AGB | Bands, VI  Topographic  Meteorological | 0.55 – 0.50 | 44.0 – 46.0 |
|  |  |  |  |  |  |  |  |  |
| ***Machine learning algorithms*** | | | |  |  |  |  |  |
| (Ramoelo et al., 2015) | South Africa | WorldView-2 | Rangelands | RF | Leaf N  Dry AGB | VI | 0.71 – 0.89  0.84 – 0.91 | 18.0 – 33.0  17.0 – 30.0 |
| (Ali et al., 2017) | Moorepark and Grange, Ireland | MODIS | Temperate grasslands | ANN  ANFIS | Dry AGB | VI, RED, NIR | 0.63-0.59  0.85-0.76 |  |
| (Wang et al., 2019) | Oklahoma, USA | Sentinel 2  Landsat 8 | Native grasslands | SVM  RF | Dry AGB | NDVI, EVI, LSWI | 0.65  0.36 |  |
| (Meng et al., 2020) | Tibetan Plateau, China | MODIS | Alpine meadow | ANN  SVM  RF | Dry AGB | Topography, atmosphere, soil and VI | 0.56  0.70  0.78 |  |
| (Reis et al., 2020) | São Paulo State, Brazil | PlanetScope | Tropical pastures intensively managed | RF  XGBoost | Dry AGB  CH  Dry AGB  CH | VI (RGB and NIR), GLCM | 0.47 - 0.60  0.86 - 0.88  0.46 - 0.65  0.86 - 0.89 | 28.4-34.4  20.9-23.7  28.4-34.4  20.9-23.7 |
| (Chabalala et al., 2020) | Mpumalanga Provinces, South Africa | Sentinel-2  RapidEye | Natural tropical grasslands | RF | Nitrogen | Bands, NDVI, SR | 0.53-0.64 | 11.3-16.0 |
| (Raab et al., 2020) | Bavaria, Germany | Sentinel-1/-2 | Temperate grasslands | RF | Dry AGB  ADF  CP  CH | S2 Bands, VI  S1 Bands | 0.45  0.79  0.72  0.60 | 24.3  6.4  12.5  19.4 |
| (Bretas et al., 2021) | Brazil | Sentinel-2  Landsat 8 | Tropical pastures *Brachiaria sp.* | RF | Fresh AGB  Dry AGB | NDVI, EVI, OSAVI, Meteorological | 0.85  0.35 |  |
| (Alvarez-Mendoza et al., 2022) | Colombia | Sentinel-2 | Tropical pastures *Brachiaria sp.* | Huber Regr.  XTree | Dry AGB | VI | 0.59  0.36 |  |
| (Dusseux et al., 2022) | France | Sentinel-2 | Temperate grasslands | BayesianRidge  ElasticNetCV  KerasRegressor  LassoCV  RidgeCV  SVR | Height | Bands, VI | 0.75  0.73  0.60  0.73  0.72  -0.03 |  |
| (Fan et al., 2022) | Tibetan Plateau, China | Sentinel-2 | Alpine meadow, alpine steppe, alpine desert | Cubist  GBRT  RF  XGBoost | Dry AGB | Bands, VI  Topographic  Meteorological | 0.62  0.83  0.88  0.83 |  |
| (Pereira et al., 2022) | Sao Paulo, Brazil | Sentinel-2  PlanetScope | Tropical pastures | RF | Dry AGB  Nitrogen  Dry AGB  Nitrogen | Bands, VI | 0.32 – 0.46  0.48 – 0.64  0.39 - 0.43  0.62 – 0.69 | 28.0 – 29.5  29.4 – 32.2  21.3 – 29.3  27.6 – 29.2 |
| (Wang et al., 2022) | Qinghai Province, China | MODIS | Alpine meadows and grasslands | RF  SVM  GBDT  ANN | Dry AGB | Bands, VI  Topographic  Meteorological | 0.58 – 0.60  0.50 – 0.55  0.56 – 0.59  0.46 – 0.53 | 40.0 – 41.0  43.0 – 45.0  40.0 – 42.0  44.0 – 47.0 |
| (Mashiane et al., 2023) | South Africa | Sentinel-2 | Mountainous grasslands | RF | Nitrogen | Bands  VI -Red Edge | 0.85 |  |
| (Bretas et al., 2023) | Mato Grosso, Brazil | Sentinel-2 | Tropical pasture | RF | Fresh AGB  CH | Bands, VI | 0.51  0.58 – 0.73 | 112.33 |
| (Vahidi et al., 2023) | Virginia, USA | Sentinel-1/-2 | Tall fescue | RF  SVR  ANN | Dry AGB | Bands, VI | 0.69  0.60  0.80 |  |
| (Zhang et al., 2023) | Qinghai-Tibet  Plateau, China | Sentinel-2  Tiangong-2 | Alpine and mountain meadows, temperate grasslands | RF  SVM | Nitrogen  Phosphorus  Potassium  Nitrogen  Phosphorus  Potassium | Bands | 0.73 – 0.76  0.69 – 0.73  0.78 – 0.81  0.67 – 0.68  0.54 – 0.61  0.74 – 0.77 | 0.30  0.03  0.39  0.34  0.04  0.44 |
| (Fernandes et al., 2024) | Sao Paulo, Brazil | Sentinel - 2 | Tropical pasture | RF  SVR | Dry AGB  Green AGB Leaf AGB  CP  NDF  Dry AGB  Green AGB Leaf AGB  CP  NDF | Bands, VI, Meteorological | 0.34  0.52  0.56  0.58  0.49  0.37  0.64  0.62  0.66  0.57 |  |
| (Guo et al., 2024) | Tibetan  Plateau, China | Sentinel-1/-2 | Alpine grassland during wilting period | RF | Dry AGB | Bands, VI, | 0.54 | 0.21 |
| (Ogungbuyi et al., 2024) | Australia | Sentinel - 2 |  | RF | Dry AGB | Bands | 0.53 - 0.58 |  |

^1^SPOT = Satellite Pour l’Observation de la Terre, MODIS = Moderate-Resolution Imaging Spectroradiometer,

^2^MLR = multiple linear regression, PLSR = partial least square regression, PLS = partial least square, ANN = artificial neural network, ANFIS = adaptive-neuro fuzzy inference systems, SVM = support vector machine, SVR = support vector regression, RF = random forest, XGBoost = extreme gradient boosting, GBRT = gradient boosting decision trees

^3^AGB = aboveground biomass, CP = crude protein, NDF = neutral detergent fiber, ADF = acid detergent fiber, CH = canopy heigh

^4^ VI = vegetation index, NIR = near infrared, NDVI = normalized difference vegetation index, GVI = tasseled Cap green vegetation index, WI = wetness index, RED = red waveband, DVI = difference vegetation index, EVI = enhanced vegetation index, SAVI = soil adjusted vegetation index, MSAVI = modified soil adjusted vegetation index, CFAPAR = seasonal cumulative fraction of the absorbed photosynthetically active radiation (FAPAR), SR = simple rate, OSAVI = optimized soil adjusted vegetation index, TSAVI = transformed soil adjusted vegetation index, ATSAVI = adjusted transformed soil adjusted vegetation index, PVI = perpendicular vegetation index, NDTI: Normalized Difference Tillage Index, LSWI = land surface water index, GLCM = grey level co-occurrence matrix, Huber Regr. = Huber regression, XTree = Extra Tree Regression

^5^ Very poor correlation between NDVI and EVI with dry AGB.

^6^ Considering the final linear models for all-year using Landsat 7 an 8. NDTI: Normalized Difference Tillage Index

**References**

Ali, I., F. Cawkwell, E. Dwyer, and S. Green. 2017. Modeling Managed Grassland Biomass Estimation by Using Multitemporal Remote Sensing Data-A Machine Learning Approach. IEEE J Sel Top Appl Earth Obs Remote Sens. 10:3254–3264. doi:10.1109/JSTARS.2016.2561618.

Alvarez-Mendoza, C. I., D. Guzman, J. Casas, M. Bastidas, J. Polanco, M. Valencia-Ortiz, F. Montenegro, J. Arango, M. Ishitani, and M. G. Selvaraj. 2022. Predictive Modeling of Above-Ground Biomass in Brachiaria Pastures from Satellite and UAV Imagery Using Machine Learning Approaches. Remote Sens (Basel). 14. doi:10.3390/rs14225870.

Askari, M. S., T. McCarthy, A. Magee, and D. J. Murphy. 2019. Evaluation of grass quality under different soil management scenarios using remote sensing techniques. Remote Sens (Basel). 11. doi:10.3390/rs11151835.

Bretas, I. L., D. S. M. Valente, T. F. de Oliveira, D. B. Montagner, V. P. B. Euclides, and F. H. M. Chizzotti. 2023. Canopy height and biomass prediction in Mombaça guinea grass pastures using satellite imagery and machine learning. Precis Agric. 24:1638–1662. doi:10.1007/s11119-023-10013-z.

Bretas, I. L., D. S. M. Valente, F. F. Silva, M. L. Chizzotti, M. F. Paulino, A. P. D’Áurea, D. S. C. Paciullo, B. C. Pedreira, and F. H. M. Chizzotti. 2021. Prediction of aboveground biomass and dry-matter content in brachiaria pastures by combining meteorological data and satellite imagery. Grass and Forage Science. 76:340–352. doi:10.1111/gfs.12517.

Chabalala, Y., E. Adam, Z. Oumar, and A. Ramoelo. 2020. Exploiting the capabilities of Sentinel-2 and RapidEye for predicting grass nitrogen across different grass communities in a protected area. Applied Geomatics. 12:379–395. doi:10.1007/s12518-020-00305-8. Available from: https://doi.org/10.1007/s12518-020-00305-8

Dusseux, P., T. Guyet, P. Pattier, V. Barbier, and H. Nicolas. 2022. Monitoring of grassland productivity using Sentinel-2 remote sensing data. International Journal of Applied Earth Observation and Geoinformation. 111. doi:10.1016/j.jag.2022.102843.

Edirisinghe, A., M. J. Hill, G. E. Donald, and M. Hyder. 2011. Quantitative mapping of pasture biomass using satellite imagery. Int J Remote Sens. 32:2699–2724. doi:10.1080/01431161003743181.

Fan, X., G. He, W. Zhang, T. Long, X. Zhang, G. Wang, G. Sun, H. Zhou, Z. Shang, D. Tian, X. Li, and X. Song. 2022. Sentinel-2 Images Based Modeling of Grassland Above-Ground Biomass Using Random Forest Algorithm: A Case Study on the Tibetan Plateau. Remote Sens (Basel). 14. doi:10.3390/rs14215321.

Fernandes, M. H. M. da R., J. de S. FernandesJunior, J. M. Adams, M. Lee, R. A. Reis, and L. O. Tedeschi. 2024. Using sentinel-2 satellite images and machine learning algorithms to predict tropical pasture forage mass, crude protein, and fiber content. Sci Rep. 14. doi:10.1038/s41598-024-59160-x.

Fernández-Habas, J., A. M. García Moreno, M. T. Hidalgo-Fernández, J. R. Leal-Murillo, B. Abellanas Oar, P. J. Gómez-Giráldez, M. P. González-Dugo, and P. Fernández-Rebollo. 2021. Investigating the potential of Sentinel-2 configuration to predict the quality of Mediterranean permanent grasslands in open woodlands. Science of the Total Environment. 791. doi:10.1016/j.scitotenv.2021.148101.

Ferreira, L. G., L. E. Fernandez, E. E. Sano, C. Field, S. B. Sousa, A. E. Arantes, and F. M. Araújo. 2013. Biophysical properties of cultivated pastures in the brazilian savanna biome: An analysis in the spatial-temporal domains based on ground and satellite data. Remote Sens (Basel). 5:307–326. doi:10.3390/rs5010307.

Friedl, M. A., J. Michaelsen, F. W. Davis, H. Walker, and D. S. Schimel. 1994. Estimating grassland biomass and leaf area index using ground and satellite data. Int J Remote Sens. 15:1401–1420. doi:10.1080/01431169408954174.

Guerini Filho, M., T. M. Kuplich, and F. L. F. D. Quadros. 2020. Estimating natural grassland biomass by vegetation indices using Sentinel 2 remote sensing data. Int J Remote Sens. 41:2861–2876. doi:10.1080/01431161.2019.1697004.

Guo, R., J. Gao, S. Fu, Y. Xiu, S. Zhang, X. Huang, Q. Feng, and T. Liang. 2024. Estimating Aboveground Biomass of Alpine Grassland During the Wilting Period Using In Situ Hyperspectral, Sentinel-2, and Sentinel-1 Data. IEEE Transactions on Geoscience and Remote Sensing. 62:1–16. doi:10.1109/TGRS.2023.3341956.

Jansen, V. S., C. A. Kolden, and H. J. Schmalz. 2018. The development of near real-time biomass and cover estimates for adaptive rangeland management using Landsat 7 and Landsat 8 surface reflectance products. Remote Sens (Basel). 10. doi:10.3390/rs10071057.

Jin, Y., X. Yang, J. Qiu, J. Li, T. Gao, Q. Wu, F. Zhao, H. Ma, H. Yu, and B. Xu. 2014. Remote sensing-based biomass estimation and its spatio-temporal variations in temperate Grassland, Northern China. Remote Sens (Basel). 6:1496–1513. doi:10.3390/rs6021496.

Mashiane, K., S. Adelabu, and A. Ramoelo. 2023. Comparative Analysis of Single Bands, Vegetation Indices, and Their Combination in Predicting Grass Species Nitrogen in a Protected Mountainous Area. Applied Sciences (Switzerland). 13. doi:10.3390/app13137960.

Meng, B., T. Liang, S. Yi, J. Yin, X. Cui, J. Ge, M. Hou, Y. Lv, and Y. Sun. 2020. Modeling Alpine Grassland Above Ground Biomass Based on Remote Sensing Data and Machine Learning Algorithm: A Case Study in East of the Tibetan Plateau, China. IEEE J Sel Top Appl Earth Obs Remote Sens. 13:2986–2995. doi:10.1109/JSTARS.2020.2999348.

Ogungbuyi, M. G., J. Guerschman, A. M. Fischer, R. A. Crabbe, I. Ara, C. Mohammed, P. Scarth, P. Tickle, J. Whitehead, and M. T. Harrison. 2024. Improvement of pasture biomass modelling using high-resolution satellite imagery and machine learning. J Environ Manage. 356. doi:10.1016/j.jenvman.2024.120564.

Pereira, F. R. da S., J. P. de Lima, R. G. Freitas, A. A. Dos Reis, L. R. do Amaral, G. K. D. A. Figueiredo, R. A. C. Lamparelli, and P. S. G. Magalhães. 2022. Nitrogen variability assessment of pasture fields under an integrated crop-livestock system using UAV, PlanetScope, and Sentinel-2 data. Comput Electron Agric. 193. doi:10.1016/j.compag.2021.106645.

Raab, C., F. Riesch, B. Tonn, B. Barrett, M. Meißner, N. Balkenhol, and J. Isselstein. 2020. Target-oriented habitat and wildlife management: estimating forage quantity and quality of semi-natural grasslands with Sentinel-1 and Sentinel-2 data. Remote Sens Ecol Conserv. 6:381–398. doi:10.1002/rse2.149.

Ramoelo, A., M. A. Cho, R. Mathieu, S. Madonsela, R. van de Kerchove, Z. Kaszta, and E. Wolff. 2015. Monitoring grass nutrients and biomass as indicators of rangeland quality and quantity using random forest modelling and WorldView-2 data. International Journal of Applied Earth Observation and Geoinformation. 43:43–54. doi:10.1016/j.jag.2014.12.010.

Reis, A. A., J. P. S. Werner, B. C. Silva, G. K. D. A. Figueiredo, J. F. G. Antunes, J. C. D. M. Esquerdo, A. C. Coutinho, R. A. C. Lamparelli, J. V. Rocha, and P. S. G. Magalhães. 2020. Monitoring pasture aboveground biomass and canopy height in an integrated crop-livestock system using textural information from planetscope imagery. Remote Sens (Basel). 12. doi:10.3390/RS12162534.

Ren, H., and G. Feng. 2015. Are soil-adjusted vegetation indices better than soil-unadjusted vegetation indices for above-ground green biomass estimation in arid and semi-arid grasslands? Grass and Forage Science. 70:611–619. doi:10.1111/gfs.12152.

Schucknecht, A., M. Meroni, F. Kayitakire, F. Rembold, and A. Boureima. 2015. Biomass estimation to support pasture management in Niger. In: International Archives of the Photogrammetry, Remote Sensing and Spatial Information Sciences - ISPRS Archives. Vol. 40. International Society for Photogrammetry and Remote Sensing. p. 109–114.

Todd, S. W., R. M. Hoffer, and D. G. Milchunas. 1998. Biomass estimation on grazed and ungrazed rangelands using spectral indices. Int J Remote Sens. 19:427–438. doi:10.1080/014311698216071.

Vahidi, M., S. Shafian, S. Thomas, and R. Maguire. 2023. Estimation of Bale Grazing and Sacrificed Pasture Biomass through the Integration of Sentinel Satellite Images and Machine Learning Techniques. Remote Sens (Basel). 15. doi:10.3390/rs15205014.

Wang, J., X. Xiao, R. Bajgain, P. Starks, J. Steiner, R. B. Doughty, and Q. Chang. 2019. Estimating leaf area index and aboveground biomass of grazing pastures using Sentinel-1, Sentinel-2 and Landsat images. ISPRS Journal of Photogrammetry and Remote Sensing. 154:189–201. doi:10.1016/j.isprsjprs.2019.06.007.

Wang, Y., R. Qin, H. Cheng, T. Liang, K. Zhang, N. Chai, J. Gao, Q. Feng, M. Hou, J. Liu, C. Liu, W. Zhang, Y. Fang, J. Huang, and F. Zhang. 2022. Can Machine Learning Algorithms Successfully Predict Grassland Aboveground Biomass? Remote Sens (Basel). 14. doi:10.3390/rs14163843.

Zhang, X., T. Liang, J. Gao, D. Zhang, J. Liu, Q. Feng, C. Wu, and Z. Wang. 2023. Mapping the forage nitrogen, phosphorus, and potassium contents of alpine grasslands by integrating Sentinel-2 and Tiangong-2 data. Plant Methods. 19. doi:10.1186/s13007-023-01024-y.
